# Replication of British Rheumatoid Arthritis Susceptibility Loci in Two Unrelated Chinese Population Groups

**DOI:** 10.1155/2013/891306

**Published:** 2013-09-03

**Authors:** Hua Li, Yonghe Hu, Tao Zhang, Yang Liu, Yantang Wang, Tai Yang, Minhui Li, Qiaoli Luo, Yu Cheng, Qiang Zou

**Affiliations:** ^1^Department of Oncology, Chengdu Military General Hospital, Chengdu 610083, China; ^2^Department of Traditional Chinese Medicine, Chengdu Military General Hospital, Chengdu 610083, China; ^3^Department of Immunology, Chengdu Medical College, Chengdu 610083, China

## Abstract

Previous genome-wide association study by WTCCC identified many susceptibility loci of common autoimmune diseases in British, including rheumatoid arthritis (RA). Because of the genetic heterogeneity of RA, it is necessary to replicate these susceptibility loci in other populations. Here, three SNPs with strong RA association signal in the British were analyzed in Han Chinese, and two SNPs (rs6457617 and rs11761231) were genotyped in the test cohort firstly. The rs6457617 was significantly associated with RA in the test cohort. The individuals bearing the homozygous genotype CC had 0.39-fold risk than these bearing the wild-type genotype TT (*P* = 0.004, OR 0.39, [95% CI 0.21–0.74]). And the protective effect of allele C was confirmed in another validation cohort with 1514 samples (*P*_*genotye* *CC*/*TT*_ = 5.9 ×  10^−10^, OR 0.34, [95% CI 0.24–0.48]). The rs6457617 can be used as a tagSNP of HLA-DQA1∗03 which encoded MHC-II **α** chain. Since MHC restriction is important for primary T-cells in positive selection and negative selection stages, MHC protein polymorphisms may be implicated in shaping the T-cell repertoire, including the emergence of a T-cell clone involved in the inflammatory arthritis.

## 1. Introduction

Rheumatoid arthritis (RA (MIM 180300)) is a common autoimmune disease characterized by chronic inflammatory, destructive, and debilitating arthritis. The etiology of RA, like that of other autoimmune disorders, is complex and remains elusive. The occurrence of RA is relatively constant with a prevalence of between 0.5 and 1.0% in several European populations [1, 2], North-American populations [3], Japan [4], and China [5]. But some native American-Indian populations have high prevalence of RA such as the Pima Indians (5.3%) [6] and Chippewa Indians (6.8%) [7]. Although it is difficult to elucidate whether the environmental or genetic effect influence the differences between populations in different countries, it is thought to have both a genetic and an environment basis [8–11]. The heritability of RA has been estimated to be about 60% [12]. The highly polymorphic HLA region is estimated to account for about one-third of the total genetic component of susceptibility [13]. Many genes outside the HLA region also account for the RA risk genetic factor.

There are two strategies used commonly to detect the RA risk genetic loci. One is the candidate gene association study, and another is the genome-wide association study (GWAS). Candidate gene association studies rely on prior knowledge of the biology of the disease and the highly subjective selection of potential genes. It is hard to find new RA susceptibility loci outside of HLA. Genome-wide association studies (GWAS) have an advantage that it entails a systematically search of the entire genome for susceptibility variants without any clue about pathogenesis. With the advent of GWAS, relatively large number of new potential susceptibility loci for RA in some populations has been identified [14, 15]. One of the most impressive GWASs was the one by the WTCCC, which scanned 500,568 SNPs (Affymetrix Chip) in 14,000 cases of seven major autoimmune diseases, including 2000 United Kingdom RA cases and 3000 controls [16]. Among the loci showing the strongest association signals, there were 3 RA susceptibility SNPs (rs6679677, rs6457617, and rs11761231) with a *P* value of less than 4 × 10^−7^. 

Meanwhile, much evidence shows that there is the genetic heterogeneity of RA across the major racial groups. For example, the haplotype in STAT4 gene associated with RA in Caucasians is not associated in the Han Chinese population, but with the presence of rheumatoid factor [17]. The rs247661 in PTPN22 gene, which was associated with RA in Europeans [18], does not exist in Han Chinese. The PADI4 gene, which was originally identified as RA susceptibility gene in large Japanese and Korean cohorts [19], was not associated with RA and unlikely to be responsible for the presence of anti-CCP autoantibodies in Caucasian populations [20]. Therefore, it is necessary to test RA susceptibility loci of GWAS in different populations. Here, the three SNPs identified by the WTCCC with a *P* value of less than 4 × 10^−7^ were taken into our consideration to investigate whether they are associated with RA in Han Chinese population.

## 2. Materials and Methods

### 2.1. Subjects Evaluated in the Association Study

Total 1894 RA patients and healthy individuals were recruited for this study. The test cohort was 380 Chengdu residents recruited from the Chengdu Medical College, and the validation cohort was 1514 Chongqing residents recruited from the Southwest Hospital, as reported in our previous study [21]. All the subjects were unrelated ethnic Han Chinese, according to self-reported ancestry. Patients have an established diagnosis of RA according to the 1987 revised criteria of the ACR for the classification of the disease. In each cohort, healthy controls were individually matched to RA cases on the basis of sex, age, ethnicity, and local residential region. There was no significant difference between the 2 cohorts in terms of the distribution of age, age at RA onset, or percentage of female subjects (Table 2). Written informed consent was obtained from all subjects, and the study was performed with the approval of the ethical committee of the Chengdu Military General Hospital.

### 2.2. SNP Selection and Genotyping Methods

Three SNPs with strong RA association signal in British population were rs6457617, rs11761231, and rs6679677. The rs6679677 has no diversity in Han Chinese population based on the HCB data of HapMap and did not genotyped in our study. The rs6457617 is in the MHC region, and the minor allele frequency (MAF) in HCB is 0.465. The rs11761231 locates on chromosome 7q32, and the MAF is 0.233. The rs6457617 and rs11761231 were genotyped using the SNPStream Ultra High Throughput Genotyping system (Beckman Coulter, Fullerton, CA, USA) according to the manufacturer's instructions. The primers and probes of each SNP were listed in Table 1. The genotype concordance rate was 100% as assessed by random retyping across different plates.

### 2.3. Statistical Analysis

Hardy-Weinberg equilibrium was assessed using a chi-square goodness-of-fit test. Case-control association analyses were performed by chi-square or Fisher's exact test, as appropriate. *P* values, odds ratios (ORs), and 95% confidence intervals (95% CIs) were calculated.

## 3. Results and Discussion

All SNPs were in Hardy-Weinberg equilibrium (*P* > 0.05) in both patients and healthy controls, indicating that our subjects were random-mating population with no selection, mutation, or migration. As shown in Table 3, rs6457617 was significantly associated with RA in the test cohort. The frequency of the minor allele C was significantly lower in RA patients compared to healthy control (28.9% of the patients versus 40.8% of the controls; *P* = 0.001, OR 0.59, [95% CI 0.44–0.80]), implying that the minor allele C was protective against RA. When we compared the distribution of the homozygous genotype CC or the heterozygous genotype CT among the RA patients and controls to the one of the wild type genotype TT, the protective effect of allele C was obvious and presented a dose effect relationship. The individuals bearing the homozygous genotype CC had 0.39-fold decreased risk than these bearing the wild-type genotype TT (*P* = 0.004, OR 0.39, [95%  CI 0.21–0.74]). while individuals bearing the heterozygous CT had 0.59-fold decreased risk for RA than those bearing wild-type genotype (*P* = 0.02, OR 0.59, [95%  CI 0.38–0.92], Table 3).

The strong association signal of rs6457617 was replicated in the validation cohort with 1514 subjects. Among 1470 samples successfully genotyped, each genotype was assessed with the use of codominant, dominant, and recessive genetic models (Table 4). The significant association was found in all genetic models. The individuals with the homozygous genotype CC had the lowest risk than those with wild-type genotype (*P* = 5.9 × 10^−10^, OR 0.34, [95% CI 0.24–0.48]). Because the allele frequencies were similar across the controls tested in both cohorts, we combined all of the data and obtained stronger statistical evidence of an association between this SNP and RA susceptibility. Compared to individuals with genotype TT, the Han Chinese bearing genotype CC had a lower decreased risk (*P* = 9.4 × 10^−12^, OR = 0.35, 95%  CI: 0.26–0.47) than heterozygous carriers had (*P* = 1.1 × 10^−4^, OR = 0.68, 95%  CI: 0.56–0.82). The allele T was RA risk allele both in the British and the Han Chinese. But the Han Chinese bearing genotype TT had a 2.86-fold (1/0.35) increased risk compared to those with genotype CC, much lower than it in the British (OR = 5.21).

This rs6457617 was identified in another GWAS in 800 Spanish subjects and replicated in an independent cohort of 794 Spanish subjects [22]. For the Asian population, this SNP was also replicated in the Korean [23]. The minor allele was C in Korean and Chinese but was T in the WTCCC British samples. With the data of each genotype frequency in RA cases and controls in Korean in their Table 3 of the previous report [23], we found that the genotype TT in Korean had a 4.77-fold increased risk compared to the Korean with genotype CC. The OR in Korean is between the one (OR 2.86) in Chinese and another (OR 5.21) in British. Recently, this SNP was replicated in another replication study in north Indians (*P* = 1.6 × 10^−9^) [24]. But this report has not shown more detail data about rs6457617. Although there are different frequencies of the alleles C and T in different populations, the association between rs6457617 and RA susceptibility exists in all these different populations, and the risk allele for RA is the same T allele, strongly suggesting that rs6457617 in HLA-DQ region is the real RA susceptibility locus.

The rs6457617 locates at 32,663,850 on 6p21.3 between the HLA-DQB1 (32,627,241-32,634,466) encoded *β* chain of MHC-II protein and HLA-DQA2 (32,709,163-32,714,664) encoded *α* chain of MHC-II protein. The MHC protein is an *α* *β* heterodimer protein receptor that is typically expressed on the surface of antigen-presenting cells. MHC loci are the most genetically variable loci in the human population. HLA DQ is highly variable, the *β* subunit more so than the *α* chain. rs6457617 can be used as a tagSNP for HLA-DQA1*03 [25, 26], which encodes MHC-II *α* chain. As we know, DRB1*0401, *0404, *0408, and *01, DQA*01, *0201, and *04 are the shared epitope (SE) associated with RA [27], while HLA-DQA1*03 has been reported to be associated with chronic HCV infection [28], type 1 diabetes [29, 30], and childhood-onset ocular myasthenia gravis [31]. Up to now, whether HLA-DQA1*03 is associated with RA has not been identified. Since rs6457617 is the RA susceptibility locus and can be a tagSNP for HLA-DQA1*03, HLA-DQA1*03 may be a SE of RA. Highly variable MHC-II molecules recognize and present different antigens to T-cells. Since MHC restriction is particularly important for primary T-cells in positive selection and negative selection stages, MHC protein polymorphisms may play an important role in shaping the T cell repertoire, including the emergence of an unusual T cell clone characterized by the potential of inflammatory arthritis. Whether the rs6457617 is a functional SNP and influences the expression or the structure of *β* chain is needed to identify. 

As for rs11761231, no association evidence was found in the allele and genotype analyses. We noticed that in the male Han Chinese, including RA patients and healthy controls, none of them bears genotype CC. The stratification of patients and controls for gender was performed. However, there is no association between rs11761231 and RA disease either in female group or in male group (Table 3). This SNP locates within the eukaryotic translation elongation factor 1 beta 2 pseudogene. Although it had very strong association signal of RA in British population, it did not show any evidence of association in Han Chinese populations. The minor allele C frequency (MAF) in Han Chinese is only 0.278, quite different from the MAF in CEU (0.407). Moreover, the prevalence of RA in China is lower than it in European. Therefore, the test cohort with 380 subjects might have no enough statistical power to detect the genetic effect of rs11761231. So, to investigate the association between rs11761231 and RA in Han Chinese, the case-control study with sufficiently large sample size is needed.

## 4. Conclusions

In this study, rs6457617 in the MHC region, which was identified as RA susceptibility locus in previous GWAS study in British, was associated with RA in the Han Chinese populations. The minor allele C has dependent protective effect of RA risk. The rs6457617 is a real RA susceptibility locus and could be a good predictor of RA risk.

## Figures and Tables

**Table 1:** PCR primers and tagged extension probes used for SNP detection.

SNPs	Locus	Gene	Primer	Sequence 5′-3′
rs6457617	6q	*MHC *	Forward	AAATGCAGTCAGTGGACTCAA
			Reverse	AAAACAAAAAAAACCCTTCAATC
			Probe	GACCTGGGTGTCGATACCTACTGTTTGTTGAGTCCATGAGCAGAT

rs11761231	7q32	*Unknown *	Forward	TGTCTTATCATGAGAACGTGCA
			Reverse	TTTTGTGTTCAAAGAATTCTGTCTT
			Probe	AGAGCGAGTGACGCATACTATGAAATCAAGAAGGTCTGAAAA

**Table 2:** Clinical characteristics of the test and validation cohorts.

Subjects	Test cohort	Validation cohort
Number of RA cases	190	757
Number of healthy controls	190	757
Study design	Unrelated cases with individually matched controls	Unrelated cases with individually matched controls
Ethnicity	Han Chinese	Han Chinese
City of residence	Chengdu	Chongqing
Female, number (%)	300 (78.9)	1253 (82.7)
Age, mean ± SD years	46.4 ± 8.0	46.8 ± 10.2
Age at RA onset, mean ± SD years*	39.4 ± 9.6	41.0 ± 10.1
Disease duration, mean ± SD years*	6.97 ± 6.2	5.7 ± 5.4

*Refers to rheumatoid arthritis (RA) patients only.

**Table 3:** Allele and genotype frequencies of SNPs in test cohort.

SNP	Chromosome	Gene	Allele	Allele analysis				Genotype	Genotype analysis			
				Controls number (%)	Cases number (%)	OR (95% CI)	*P*		Controls number (%)	Cases number (%)	OR (95% CI)	*P*
rs6457617	6q	MHC	T	218 (59.2)	263 (71.1)	1 (reference)	—	TT	68 (37.0)	97 (52.4)	1 (reference)	—
			C	150 (40.8)	107 (28.9)	**0.59 (0.44**–**0.80)**	**0.001**	CT	82 (44.6)	69 (37.3)	**0.59 (0.38–0.92)**	**0.02**
								CC	34 (18.5)	19 (10.3)	**0.39 (0.21–0.74)**	**0.004**

rs11761231	7q32	Unknown	T	287 (75.9)	290 (76.7)	1 (reference)	—	TT	108 (57.1)	112 (59.3)	1 (reference)	—
			C	91 (24.1)	88 (23.3)	0.96 (0.68–1.34)	0.80	CT	71 (37.6)	66 (34.9)	0.90 (0.59–1.37)	0.62
								CC	10 (5.3)	11 (5.8)	1.06 (0.43–2.60)	0.90

rs11761231 in female group	7q32	Unknown	T	218 (73.2)	220 (73.8)	1 (reference)	—	TT	79 (53.0)	82 (55.0)	1 (reference)	—
			C	80 (26.8)	78 (26.2)	0.97 (0.67–1.39)	0.85	CT	60 (40.3)	56 (37.6)	0.90 (0.56–1.45)	0.66
								CC	10 (6.7)	11 (7.4)	1.06 (0.43–2.63)	0.90

rs11761231 in male group	7q32	Unknown	T	69 (86.3)	70 (87.5)	1 (reference)	—	TT	29 (72.5)	30 (75.0)	1 (reference)	—
			C	11 (13.7)	10 (12.5)	0.90 (0.36–2.25)	0.82	CT	11 (27.5)	10 (25.0)	1.14 (0.42–3.08)	1.0
								CC	0	0		

Data shown in *n* (%). OR = odds ratio; 95% CI = 95% confidence interval; MHC: major histocompatibility complex; allele analyses for association were used by Pearson chi-square test, and genotype analyses for association were used by logistic regression.

**Table 4:** Validation of SNPs in test cohort, validation cohort, and combined analyses (combined validation cohort with test cohort).

	Genotype	Subjects number (%)		Codominant genetic model		Dominant genetic model		Recessive genetic model	
		Controls	Cases	OR (95% CI)	*P*	OR (95% CI)	*P*	OR (95% CI)	*P*
Test cohort rs6457617	TT	68 (37.0)	97 (52.4)	1 (ref)	—	**0.53 (0.35–0.81)**	**0.003**	**0.51 (0.28–0.92)**	**0.025**
	CT	82 (44.6)	69 (37.3)	**0.59 (0.38–0.92)**	**0.02**				
	CC	34 (18.5)	19 (10.3)	**0.39 (0.21–0.74)**	**0.004**				

Validation cohort rs6457617	TT	263 (35.7)	353 (48.2)	1 (ref)	—	**0.60 (0.49–0.74)**	1.5 × 10^−6^	**0.41 (0.30–0.56)**	2.3 × 10^−8^
	CT	340 (46.1)	319 (43.5)	**0.70 (0.56–0.87)**	1.0 × 10^−3^				
	CC	134 (18.2)	61 (8.3)	**0.34 (0.24–0.48)**	5.9 × 10^−10^				

Combined samples rs6457617	TT	331 (35.9)	450 (49.0)	1 (ref)	—	**0.58 (0.48–0.70)**	1.4 × 10^−8^	**0.43 (0.32–0.57)**	2.2 × 10^−9^
	CT	422 (45.8)	388 (42.3)	**0.68 (0.56–0.82)**	1.1 × 10^−4^				
	CC	168 (18.3)	80 (8.7)	**0.35 (0.26–0.47)**	9.4 × 10^−12^				

Data shown in *n* (%). OR = odds ratio; 95% CI = 95% confidence interval; allele analysis and genotype analysis in dominant genetic model or in recessive genetic model for association were used by Pearson chi-square test, and genotype analyses in codominant genetic model for association were used by logistic regression.
